# Life Cycle Assessment (LCA) of Bioplastics Production from Lignocellulosic Waste (Study Case: PLA and PHB)

**DOI:** 10.3390/polym16233330

**Published:** 2024-11-27

**Authors:** Lacrimioara Senila, Eniko Kovacs, Maria-Alexandra Resz, Marin Senila, Anca Becze, Cecilia Roman

**Affiliations:** 1 Research Institute for Analytical Instrumentation Subsidiary, National Institute for Research and Development of Optoelectronics Bucharest INOE 2000, 67 Donath Street, 400293 Cluj-Napoca, Romania; lacri.senila@icia.ro (L.S.); anca.becze@icia.ro (A.B.); cecilia.roman@icia.ro (C.R.); 2 Research Institute for Organic Auxiliary Products, 551022 Medias, Romania; M.AlexandraResz@gmail.com

**Keywords:** life cycle assessment, bioplastics, orchard waste, L-polylactic acid, poly(3-hydroxybutyrate)

## Abstract

Life cycle assessment of a technology is the key to technological development in the context of sustainable development. Orchard waste has been identified as a potential source of bioplastics. The objective of this study was to conduct a life cycle assessment of two specific bioplastic materials, namely, L-polylactic acid (PLA) and poly(3-hydroxybutyrate) (PHB). Bioplastics, such as PLA acid and PHB, can be used as alternatives to conventional plastics due to their biodegradability and non-toxicity, both of which have the potential to replace conventional petroleum-based plastics. Polylactic acid was synthesized from orchard waste in a series of stages, including biomass processing, pretreatment for carbohydrate extraction, simultaneous saccharification and fermentation (SSF), and microwave polymerization. PHB, another biodegradable polymer, is produced by microorganisms through the fermentation of sugars obtained from the same biomass. Applied LCAs show that for PLA production, the stages having the greatest environmental impact are biomass processing, pretreatment, and the SSF process, and for PHB production, very energy-intensive stages significantly contributing to the environmental impacts are biomass processing and pretreatment stages. For both PLA and PHB, the initial stages of biomass processing and pretreatment are the most energy-intensive and significant contributors to CO_2_ emissions.

## 1. Introduction

The cultivation of fruit trees represents a significant aspect of agricultural activity around the world and in Romania. Significant quantities of lignocellulosic waste are generated on an annual basis. At present, there are various disposal techniques employed, including the storage of waste in the field, the incorporation of shredded chips into the soil, and field burning. The production of bioplastics from orchard waste represents an environmentally sustainable solution to the problem of waste disposal, avoiding the emission of greenhouse gases. Agricultural waste is treated for recovery instead of land use or disposal, creating a new circular process in a given linear production context. Waste management and biodegradation are issues of national and international concern. It is imperative that biodegradable materials are produced in such a way that they degrade rapidly without contaminating soil, air, and groundwater [1,2,3]. The obtained bioplastics are used for plasticization, copolymerization, and melt blending with different tough polymers, rubbers, thermoplastic elastomers, and nanomaterials [4]. Accordingly, the proposed method entails the fabrication of bioplastics, including L-polylactic acid (PLA) and poly(3-hydroxybutyrate) (PHB), from biomass. Bioplastics are produced from polymers, separated from biomass by using specific methods. While bioplastic has the potential to replace conventional plastic, concerns remain regarding its environmental impact. These include greenhouse gas emissions and the negative effects on land use [5].

The production of green plastics, such as polyhydroxyalkanoates (PHAs), has a favorable impact on the global market. However, the environmental sustainability and life cycle assessment of this type of technology for the production of green plastics are not yet fully understood [6]. Biobased polymers used as bioplastics are divided into the following three classes: plant-based (thermoplastic starch, TPS), polymerized biomonomers (PLAs), and extracted biopolymers (PHBs) [7]. PLA is a thermoplastic polyester derived from renewable resources; it is very easy to use and has a rapid degradation rate, typically less than 2 years. Polyhydroxyalkanoates represent a class of biodegradable and renewable bioplastics that share certain properties similar to traditional plastics such as polyethylene (PE) and polypropylene (PP) [8]. However, they offer advantages such as biodegradability and renewability. Depending on the R-hydroxyalkanoic acid monomer unit, PHAs are divided into the following three types: short-chain-length PHA (scl-PHA), medium-chain-length PHA (mcl-PHA), and long-chain-length PHA (lcl-PHA) [9]. Poly(3-hydroxybutyrate) [P(3HB)] and poly(3-hydroxybutyrate-co-3-hydroxyvalerate) [P(3HB-co-3HV)] are the most important polymers with multiple applications, including packaging, biomedicine, agriculture, textiles, and electronics [10]. Polylactic acid is the most significant polymer in terms of production volume, with an output of 460 thousand tons and 180,000 tons of production per year. In general, polylactic acid is produced from lactic acid [11,12]. The production of polylactic acid from biomass involves the following technological steps: pre-treatment of biomass, hydrolysis and fermentation to lactic acid, and polymerization of lactic acid to polylactic acid. Polylactic acid is a well-known biodegradable bioplastic that has the potential to replace traditional plastics such as PP, PE, and acrylonitrile butadiene styrene. PLA is a thermoplastic polymer that can have three different stoichiometric forms, namely, poly-L-lactide (PLLA), poly-D-lactide (PDLA), and poly-D,L-lactide (PDLLA) [13]. These forms can be obtained through the polymerization of lactic acid produced by a fermentation process [13]. By the decomposition of biodegradable PLA, carbon dioxide and water can be produced [14]. In our previous study, PLA production was carried out by microwave irradiation of lactic acid obtained from lignocellulosic biomass [15]. The production of virgin PLA required a large amount of energy, depending on the technological steps. The production of eco-friendly technologies from waste through biowaste valorization, resulting in the production of bioplastics, is an important step towards the implementation of a circular economy. Furthermore, reforming bioplastic production represents a key factor in achieving sustainability [16]. PLA and PHB are the most prominent examples of biodegradable, bio-based plastics produced through the fermentation of lignocellulosic waste using specific bacteria. The proportion of D- or L-isomers determines the degree of crystallinity of PLA, which influences its thermal stability and mechanical properties. Pure L-isomer has higher mechanical strength than mixtures of D- and L-isomers. Both PLA and PHB are biodegradable bioplastic alternatives. They are derived from renewable resources, have a reduced environmental impact, and can reduce the environmental footprint of plastic products. PHB is fully biodegradable and can decompose in the natural environment. The production of PLA and PHB results in lower greenhouse gas emissions than those associated with the production of conventional plastics while having similar properties [17].

Some studies have been conducted on the life cycle assessment (LCA) of bioplastic production from biomass. The LCA approach has been used to measure the environmental impacts associated with the production of materials along the entire value chain [18,19,20]. According to Osman et al. (2024), the LCA approach evaluates products throughout their entire life cycle, from production to disposal. The establishment of system boundaries is based on different options, such as cradle-to-gate, cradle-to-grave, gate-to-gate, and grave-to-grave [21]. The cradle-to-grave system takes into account all the technological stages and the entire life cycle.

In a recent study, Li et al. (2024) presented a life cycle assessment and artificial intelligence model for uncertainty analysis of PLA production from corn stover. The model employed a cradle-to-gate LCA analysis-based conversion process that included pre-treatment, enzymatic hydrolysis, fermentation, purification, and polymerization. Three scenarios were considered with regard to the end-of-life cases and fuel options. The results showed that for 1 kg of PLA, 4.3 kg CO_2_ eq is required for composting PLA with natural gas, 3.7 kg CO_2_ eq for incineration of PLA with natural gas, and 1.9 kg CO_2_ eq for incineration of PLA with wood pellets for electricity generation [22]. A review of bioplastic production from various renewable sources in the context of LCA, the negative impacts of fossil-based plastics, and the advantages of bioplastic production from renewable sources was published by Ali et al. (2023) [16].

Rebolledo-Leiva et al. (2023) employed the LCA methodology to investigate the environmental impact of polylactic acid derived from wheat. Their findings show that the fermentation process used for the production of lactic acid is a significant contributor to global warming, with a range of 1.38 to 0.44 kg CO_2_ eq [11]. Finding new sources for the production of biodegradable polymers represents a major opportunity to reduce environmental pollution. According to Lyu et al. (2023), the substitution of polypropylene with biodegradable polylactic acid generates benefits and reduces emissions by 37% [10]. Also, Kovacs et al. (2020) performed a life cycle assessment of biofuel production from viticultural wastes and quantified human toxicity, global warming, fine particulate matter formation, terrestrial ecotoxicity, fossil resource scarcity, water consumption, freshwater eutrophication, and freshwater ecotoxicity [23]. An environmental life cycle assessment of polyhydroxyalkanoate production by purple phototrophic bacteria mixed cultures was conducted using two scenarios: the first involved the extraction of PHA, while the second focused on anaerobic digestion in a combined heat and power plant (CHP) [7]. Among all contributors, the photobiorefinery fermentation stage has the greatest impact on the environment due to the direct emission and consumption of electricity. Vea et al. (2021) studied the impact categories of LCA for polyhydroxyalkanoate (PHA)-based plastics production and reported that the production of plastics based on plants bacterial fermentation has a higher environmental impact in comparison to conventional plastics [24].

Aryan et al. (2021) conducted a comparative life cycle assessment of various PLA valorization scenarios: production of lactic acid, methyl lactate, and ethyl lactate, and incineration with the production of electricity and heat. The possibility to recover the solvents used for PLA valorization with the generation of by-products can have higher environmental benefits compared to the incineration scenario [20]. In the literature, among the renewable resources used for the production of PLA, corn is the most widely used, followed by sugarcane [25]. Ioannidou et al. (2022) used LCA and life cycle costing (LCC) to evaluate the sustainability potential of PLA production from corn stover, corn glucose syrup, and sugar beet pulp [26]. Through an LCA study, Morão et al. (2019) identified the environmental impacts of PLA production from sugarcane [27]. Riofrio et al. (2022) studied the environmental viability of the production process of PLA from Ecuadorian sugarcane in order to identify the stages that have a positive or a negative effect on the environment, and also by comparison with synthetic polymers [28]. The literature on the production of PHB employing the LCA methodology is very limited. Research was found on the production of PHB from soybean oil and sucrose [29], from by-products derived from sunflower-based biodiesel [30], and from rapeseed oil derivatives [31].

The objective of this study was to evaluate the environmental impact of two bioplastic production technologies through the employment of the life cycle assessment (LCA) methodology, namely polylactic acid (PLA) and poly(3-hydroxybutyrate) (PHB) derived from lignocellulosic biomass. The analysis focused on evaluating the environmental impacts of each technology throughout the various stages of its life cycle, comparing their respective environmental performances, and identifying the key stages that contribute to the overall impacts.

## 2. Materials and Methods

### 2.1. Technology for PLA Production from Lignocellulosic Biomass

The procurement of biomass represents a fundamental stage in the LCA of bioplastic production technologies. The biomass waste was collected from the Research Station of the University of Agricultural Sciences “Ion Ionescu de la Brad” in Iasi, Romania. The samples were dried at 105 °C and shredded to a diameter of 0.2 mm.

The technology for PLA production was presented in our recent open-source paper [15]. PLA production takes place in four stages: pressurized hot water pretreatment for hemicellulose separation, simultaneous saccharification and fermentation process of solid fraction (enzymatic hydrolysis and fermentation occur simultaneously) directly to lactic acid, lactic acid purification, polymerization under microwave irradiation (first water was removed by evaporation to obtain lactide and microwave irradiation directly to PLA), and PLA purification. The composition of plum wastes was 38.4% cellulose, 26.8% hemicellulose, 28.6% lignin, 3.8% ash, and 2.4% others.

#### 2.1.1. Biomass Process

The biomass was subjected to grinding and homogenization prior to the pretreatment process under high pressure. The first stage consists of the collection of biomass, its transportation, storage, and natural drying. Subsequently, preliminary processing, including grinding and homogenization, was carried out. The samples were subjected to drying at 105 °C and shredding to a diameter of 0.2 mm prior to processing into bioplastics.

#### 2.1.2. Pretreatment

The pressurized hot water of biomass was conducted in a Parr reactor (Parr Instruments, Moline, IL, USA) by the addition of dry biomass and water (1:7 *w*/*w*), which was then subjected to a 30 min reaction at 180 °C and 100 bars. The phases were subsequently separated, and the solid phase was subjected to the SSF process, enzymatic hydrolysis, and fermentation to lactic acid production (48 h).

#### 2.1.3. SSF Process

The solid fraction resulted after pretreatment was employed in the subsequent SSF process. The fermentation process was carried out in a 1.7 L bioreactor (Lambda Minifor, Lambda Laboratory Instruments, Brno, Czech Republic). The mixtures of the pretreated biomass (10%), nutrients, cellulase from *Trichoderma reesei* ATCC 26,921 (25 FPU/g), β-glucosidase from almonds (20 U/g), and inoculum of *L. rhamnosus* (10%) were mixed at 37 °C for 72 h [15]. Following the fermentation process, lactic acid and a residual fraction of the culture medium were obtained. Subsequently, the lactic acid was subjected to further separation and purification.

#### 2.1.4. Lactic Acid Purification

The lactic acid was extracted from the fermentation broth by liquid–liquid extraction with ammonium sulfate and butanol. The organic phase (rich in lactic acid) was then separated and subjected to vacuum drying in order to remove any remaining moisture. The resulting waste products were butanol and wastewater.

#### 2.1.5. Polymerization of Lactic Acid to PLA

PLA was obtained after the polymerization of the lactic acid by microwave irradiation at 140 °C for 30 min using o-xylene as solvent and SnCl_2_ as catalyst (0.4 wt.%) (first water was removed by evaporation to obtain lactide and microwave irradiation directly to PLA), followed by PLA purification.

### 2.2. Technology for PHB Production from Lignocellulosic Biomass

The technology for PHB production from lignocellulosic biomass was presented in our recent paper [19]. The apple orchard biomass contains 40.0% cellulose, 21.95% hemicellulose, and 29.62% lignin. The technology is composed of the following stages: microwave pretreatment, ammonia delignification, enzymatic hydrolysis, and fermentation using the *Bacillus megaterium* ATCC 14581 strain. The pretreated biomass was delignified with 20% (*w*/*v*) ammonia for 12 h at 80 °C, followed by enzymatic hydrolysis using cellulase enzymes at 50 °C for 72 h.

#### 2.2.1. Biomass Process

The biomass processing stages prior to the conversion to PHB were identical to those employed in the production of PLA.

#### 2.2.2. Microwave Irradiation

The biomass was treated by a microwave-assisted digestion system (MWS-3+, Berghof Instruments, Eningen, Germany) operating at 190 °C for a period of 30 min. A microwave power of 700 W was employed during this process. After microwave irradiation, the samples were collected and subsequently separated. The solid fraction, containing cellulose and lignin, was separated by filtration.

#### 2.2.3. Delignification with Ammonia

The pretreated biomass was delignified with 20% ammonia for 12 h at 80 °C. The solid fraction was dried and used for enzymatic hydrolysis. The output products of this process were lignin and wastewater.

#### 2.2.4. Enzymatic Hydrolysis

The delignified biomass was subjected to hydrolysis with a mixture of cellulase enzymes (*Trichoderma reesei* ATCC 26921 and *β-glucosidase*) in citrate buffer (0.05M, pH = 4.8) at 50 °C for 72 h. The resulting glucose solution was then used for fermentation with *Bacillus megaterium* ATCC 14581.

#### 2.2.5. Fermentation

The hydrolysates were fermented with *Bacillus megaterium* bacteria in a 1.7 L bioreactor (Lambda Minifor, Lambda Laboratory Instruments, Brno, Czech Republic). The inoculum of bacteria was grown separately in a liquid nutrient broth for 8 h at 35 °C. The fermentation experiments were conducted at 35 °C for 48 h. The output products were PHB and fermentation waste.

#### 2.2.6. PHB Separation

The PHB was extracted from the fermentation broth by reacting it with chloroform at 100 °C for 1 h. Following the evaporation of the solvent, the pellets of PHB were separated.

### 2.3. LCA of PLA and PHB Obtained from Lignocellulosic Biomass

#### 2.3.1. Goal and Scope

Life cycle assessment is a method by which the consumption of energy and materials and the impact on human health and ecosystems are evaluated in detail for each stage of the technological process. The current LCA study is based on the ISO 14040 and ISO 14044 standardized methodology, consisting of the following four phases [32,33]: a goal and scoping phase to define the objective, the functional unit, and the boundaries of the system; an inventory phase to quantify the materials, including the inputs and outputs of all the processes involved; an impact assessment to map the potential impacts of the unit flows to category indicators and impact factors; and finally, an interpretation phase to discuss the results obtained. All the abovementioned phases are presented in the following sections.

**Figure 1:**
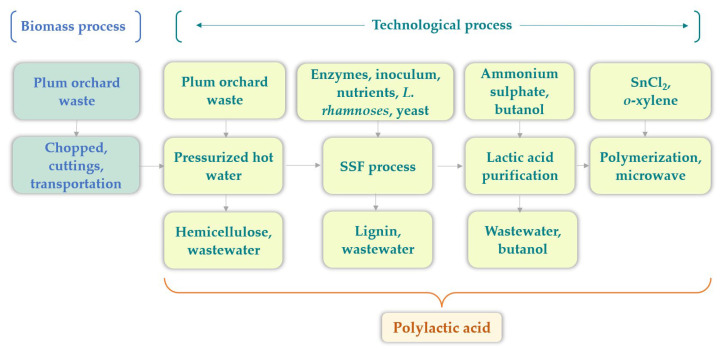
PLA production from lignocellulosic biomass.

The goal of the current LCA was to evaluate the environmental impacts of PLA and PHB production technologies from lignocellulosic biomass. The scope, including the product system and the system boundary, for the production of PLA from plum orchard waste is shown graphically in Figure 1. In the case studies, a cradle-to-gate approach is used for both the PLA and PHB. The system boundary includes all process inputs and emissions associated with the two technologies under study, and these are tracked within the LCA. Inputs within and between each production step were considered. An attributional model was used in the current study. The functional unit for the case studies was defined as the treatment of 100 g of lignocellulosic biomass in order to obtain PLA and PHB, resulting in 26.8 g of PLA and 7.3 g of PHB. This functional unit enables the comparison of the two products.

**Figure 2:**
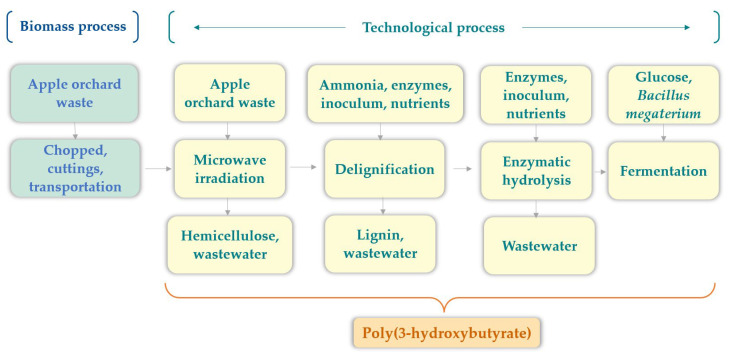
PHB production from lignocellulosic biomass.

The scope of the PHB production from apple orchard waste is represented in Figure 2.

The primary data used for the inventory was derived from the PLA and PHB production processes at a laboratory scale and from the Ecoinvent database. Regarding the impact assessment, the Impact 2002+ method was applied using the professional software SimaPro v. 9.0. The mass input and output flows were considered in the LCA process.

#### 2.3.2. Life Cycle Inventory (LCI)

The initial stage of the process involves the milling of plum orchard waste, which results from the tree pruning, which requires electricity sourced from the grid. The resulting biomass was then transported to the laboratory for pretreatment in order to separate the cellulose. The cellulose, along with lignin, was then fermented to produce lactic acid, which was subsequently used to synthesize PLA through microwave polymerization. The transport, collection, and grinding of biomass were considered before the treatment of the biomass. The energy required for the biomass pretreatment (i.e., for increasing the temperature of the reaction mixture, applying pressure, and maintaining the temperature for the required time) was evaluated theoretically. Also, the electricity needs for the SSF and microwave processes were calculated theoretically. The life cycle inventory for the production of PLA is presented in Table 1.

The process employed to obtain PHB from the lignocellulosic biomass comprises five principal stages, namely, (a) the biomass pretreatment stage, (b) the delignification stage, (c) the enzymatic hydrolysis stage, (d) the fermentation stage, and (e) the stage of separating PHB into chloroform. Table 2 presents the life cycle inventory for the production of PHB.

In the LCA applied for the PLA production, assumptions related to the energy sources were made, such as “Electricity, medium voltage {RO}| market for”, and the associated processes, such as “Transmission network, electricity, high voltage {CA-QC}| transmission network construction, electricity, high voltage”, and the sources of the chemical substances used in the experiments, namely, “Ammonium sulfate, as N {GLO}| market for” and “Copper, from solvent-extraction electro-winning {GLO}| copper production”, the used fossil fuels for transportation involved in obtaining the resources, such as “Petroleum {RoW}| petroleum and gas production”, “Diesel, from crude oil, consumption mix, at refinery, 200 ppm sulfur EU-15”, and “Natural gas, high pressure {NL}| petroleum and gas production”, and the transportation type involved in all processes that were taken into account for the PLA LCA: “Small lorry transport, Euro 0, 1, 2, 3, 4 mix, 7.5 t total weight, 3.3 t max payload”, “Transport, pipeline, long distance, natural gas {RER w/o DE+NL+RU}| transport, pipeline, long distance, natural gas”, and the discharged waste and involved processes after applying industrial processes, such as “Municipal solid waste {GLO}| treatment of municipal solid waste”, “Waste plastic, mixture {GLO}| treatment of waste plastic, mixture, open burning”, and “Waste gypsum {Europe without Switzerland}| treatment of waste gypsum, sanitary landfill”. However, in the LCA applied for the PHB production, assumptions were made in the case of energy sources (electricity, high voltage {RO}| electricity production; diesel, burned in diesel-electric generating set, 10MW; heat, district or industrial, other than natural gas {RoW}; Sulfur {RoW}| natural gas production; wind turbine network connection, 2MW, and onshore), transportation (small lorry transport, Euro 0, 1, 2, 3, 4 mix, 7.5 t total, transport, freight train {RoW}| diesel; transmission network, electricity, medium voltage; and railway track {RoW}| construction), sources of the chemical substances (sodium hydroxide, without water, in 50% solution state), and discharged waste (waste natural gas, sour {GLO}| treatment; waste gypsum {RoW}| treatment of waste gypsum; municipal solid waste {GLO}| treatment of municipal; and water discharge from petroleum/natural gas extraction).

#### 2.3.3. Life Cycle Impact Assessment (LCIA)

In order to obtain a comprehensive understanding of the environmental impact of the PLA and PHB production processes, a detailed analysis was conducted, encompassing all impact categories specific to the Impact 2002+ method. Thus, the assessment of the environmental performance was conducted in 15 categories of environmental and human health impacts quantified by using the midpoint indicators in the Impact 2002+ method. These are as follows: terrestrial ecotoxicity (eq kg triethylene glycol and TEG in soil), terrestrial acidification/nutrification (eq kg SO_2_), aquatic acidification (eq kg SO_2_), aquatic ecotoxicity (eq kg triethylene glycol and TEG in water), aquatic eutrophication (kg PO_4_^-^P limit), global warming (eq kg CO_2_), ozone depletion (eq kg trichlorofluoromethane, CFC-11), non-renewable energy (eq kg primary MJ), mineral extraction (surplus MJ), land use (m^2^ of organic farm land), respiratory inorganic substances (eq kg PM2.5), respiratory organic substances (eq kg C_2_H_4_), non-carcinogenic substances (eq kg C_2_H_3_Cl), carcinogenic substances (eq kg C_2_H_3_Cl), and ionizing radiation (Bq C-14 eq). Eutrophication was caused by an abundance of nutrients. Aquatic acidification was the result of the exposure to acidifying compounds. The ozone depletion was determined by human toxicity due to the elimination of chemicals into freshwater. The estimated impact is grouped into seven environmental categories: (1) climate change: global warming potential (GW); (2) air pollution: ozone layer depletion (ODP), respiratory inorganics; (3) water and soil pollution: aquatic eutrophication (AE), terrestrial acid/nutrification potential (TAP), aquatic acidification; (4) ecotoxicity: terrestrial ecotoxicity, aquatic ecotoxicity; (5) land use: land occupation; (6) human health: carcinogens, non-carcinogen categories, and ionizing radiation; and (7) resource depletion: mineral extraction and non-renewable energy.

## 3. Results and Discussion

The LCA results present the environmental impacts of each technology stage, such as biomass processing, pretreatment of biomass, SSF processing, lactic acid purification, and microwave irradiation for PLA production. The environmental profile of PLA production from lignocellulosic waste (for 26.8 kg PLA) is presented in Table 3.

The environmental impact categories identified as having the most significant impact related to the PLA production were non-renewable energy, global warming, aquatic ecotoxicity, ionizing radiation, terrestrial ecotoxicity, and terrestrial acidification/nutrification. In the case of PLA production, non-renewable energy is consumed primarily in biomass processing and pretreatment, where energy is required to break down lignocellulosic biomass into fermentable sugars and the polymerization process, including the energy-intensive steps involved in microwave polymerization. The pretreatment process has the highest aquatic ecotoxicity (28,497.1 kg TEG water) due to the large amount of water discharged. The first stage of the biomass process contributes significantly to the total PLA production, in line with other studies [34].

The pretreatment has the greatest impact of all types due to the electricity and heat generated. Greenhouse gas (GHG) emissions result from the direct combustion of biomass, which releases the biogenic carbon contained in PLA into the environment. Pretreatment has the highest CO_2_ content (8990.5 kg CO_2_ eq), followed by biomass processing, the SSF process, and a small amount from microwave irradiation. This is due to the high energy required to achieve the high temperature and pressure. Large amounts of waste are eliminated. The SSF process significantly reduces the need for electricity. Global warming from the biomass process is mainly due to the processing of the biomass (cutting, transport, and drying) before the technology is applied for PLA production. The SSF process has a moderate impact on GW due to the use of nutrients, enzymes, and yeast (but non-toxic chemicals). Lactic acid purification has a lower impact on aquatic ecotoxicity and the production of non-renewable energy due to the use of solvents for extraction. The GWP emission of 26.8 kg of polymer was 512.3 kg/ton. The GHG emission for PLA production is net positive compared to the GHG emission for synthetic plastics (almost 3500 kg CO_2_ eq ton of polymer) [35]. Morao and de Bie (2019), in a LCA study conducted on the production of PLA from sugarcane, also found that chemicals and energy use had the greatest environmental impact, particularly during the lactic acid production stage [27]. Ozone depletion, aquatic eutrophication, and mineral extraction have an insignificant contribution. Aquatic ecotoxicity contains the potential toxic substance release during PLA production (reagent release during pretreatment and purification stages). Additionally, wastewater generated during the fermentation and purification processes may contain contaminants that contribute to aquatic ecotoxicity if not treated adequately. In regard to human toxicity (carcinogenic and non-carcinogenic), the contribution of the technological processes utilized for PLA production is expressed in kg C₂H₃Cl eq. The non-carcinogenic human toxicity contributes with 6.3 kg C_2_H_3_Cl eq (pretreatment stage) and with 2.3 kg in the biomass processing stage. The cumulative contribution of all process steps for 26.8 kg of PLA is less than 4.5 kg of C_2_H_3_Cl equivalents per kg of PLA. It can be concluded that the agricultural stage and biomass pretreatment are major contributors to carcinogenic human toxicity and freshwater eutrophication during the PLA production process.

Terrestrial acidification/nutrification generates SO_2_ emissions associated with sulfuric acid production. PLA production was found to have disadvantages in some categories, such as ionizing radiation, acidification, and aquatic toxicity. The non-renewable energy production for 1 ton of PLA is 7399.99 MJ/ton of polymer, which is lower compared to 1 ton of synthetic polymer. The cost of PLA production has been reduced by using biowaste. In the SSF process, *Lactobacillus* sp. was used for the fermentation of sugars to lactate. In general, enzymatic hydrolysis and fermentation occur as separate processes, but the combination of both processes (enzymatic hydrolysis and fermentation) significantly reduces the GWP. In the human carcinogenic toxicity impact category, the following substances have been identified by the SimaPro v. 9.0 software: electricity, natural gas, polyvinyl chloride waste, ammonium sulfate, iron production, high-voltage electricity, copper, sintering, heavy coal ash, xylene, and iron. The total impact is represented by the pretreatment stage (2.85 kg C_2_H_3_Cl eq), biomass processing (1.11 kg C_2_H_3_Cl eq), the SSF process (0.4 kg C_2_H_3_Cl eq), polymerization (0.08 kg C_2_H_3_Cl eq), and lactic acid purification (0.802 kg C_2_H_3_Cl eq).

Mediboyina et al. (2024) conducted a life cycle assessment for lactic acid production from dairy side streams by using a cradle-to-gate approach and reported a 23.98 kg CO_2_ eq. kg^−1^ climate change impact [36]. Other renewable sources of PLA investigated using LCA include corn stover, corn glucose syrup, and sugar beet pulp. The highest GWP of the production process of 1 kg PLA was associated with the conversion of sugar beet pulp (2.25 kg CO_2_-eq/kg_PLA_), followed by corn stover (1.04 kg CO_2_-eq/kg_PLA_) and glucose syrup (0.95 kg CO_2_-eq/kg_PLA_) [28]. In the case of sugarcane bagasse conversion to lactic acid, the pretreatment and fermentation stages had the greatest impact on climate change (4.62 kg CO_2_ eq kg^−1^) [37].

**Figure 3:**
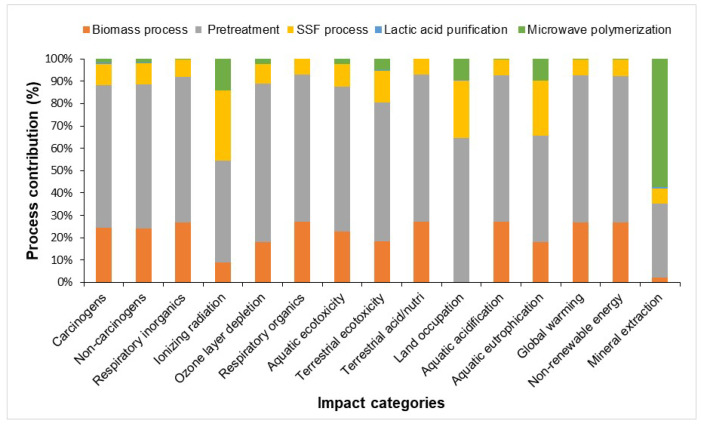
Process contributions of each impact category for the life cycle of PLA.

The normalized contribution, expressed in percentage, of the impact factors, depending on each technology step involved in PLA production from plum orchard waste, calculated by the Impact 2002+ method in the SimaPro software is presented in Figure 3.

**Figure 4:**
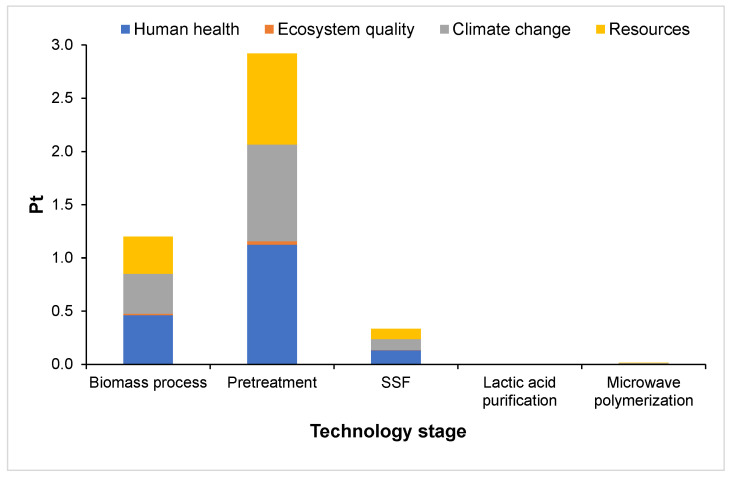
Damage impact depending on the technology stage for PLA production.

The damage categories identified are human health, ecosystem quality, climate change, and resources. The impact of damage categories depending on the technology stages is presented in Figure 4. During plastics production, carbon monoxide, dioxins, nitrogen oxides, and hydrogen cyanide are some compounds that are released in the air [16].

The production of PLA from lignocellulosic biomass represents an emerging technology with significant economic implications. The implementation of this technology would have a significant impact on the following aspects: reduction of feedstock costs, stimulation of rural economies, enhanced economic resilience, adherence to environmental regulations, market opportunities for environmentally friendly products, and innovation and technology development.

**Figure 5:**
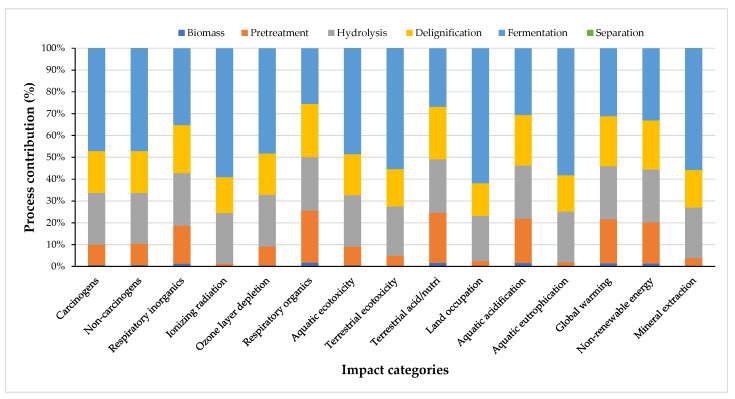
Process contributions of each impact category for the life cycle of PHB.

The PHB production involves the following stages: biomass processing, biomass pretreatment, delignification, enzymatic hydrolysis, fermentation, and PHB purification. The environmental impact categories associated with each stage are presented in Table 4. Figure 5 presents the process contributions of each impact category for the life cycle of PHB production. 

**Figure 6:**
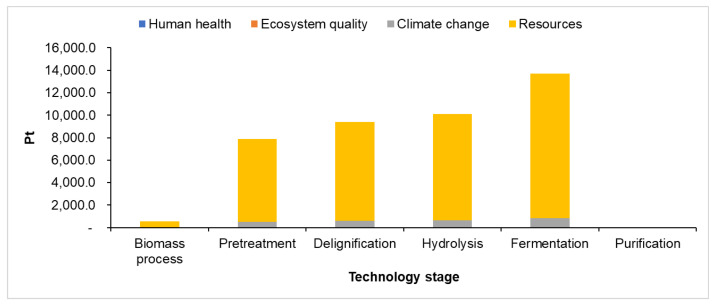
Damage impact depending on the technology stage for PHB production.

The potential damage impact associated with the various stages of PHB production technology is illustrated in Figure 6.

The fermentation process has the greatest influence on the environmental impact of technology, across all impact categories. Fermentation typically requires the utilization of extensive electricity for the maintenance of specific bioreactor conditions, including temperature, agitation, and aeration. Additionally, the fermentation stage may involve the consumption of significant amounts of chemicals (such as nutrients or pH stabilizers) during the microbial conversion of sugars into PHB.

The culture medium was prepared by weighing nutrients, minerals, and bacteria that facilitate growth under specific conditions. The categories of impact that exert a considerable effect are non-renewable energy, ionizing radiation, terrestrial ecotoxicity, aquatic ecotoxicity, and global warming. The production of 7.3 kg of PHA resulted in a significant contribution to the environmental impact associated with the utilization of non-renewable energy (38,995.5 MJ primary), ionizing radiation (21,015.05 kBq Co-14 eq), aquatic ecotoxicity (18,919.97 kg TEG water), terrestrial ecotoxicity (3693.62 kg TEG soil), and global warming (2520.82 kg CO₂ eq).

Non-renewable energy consumption is a critical factor in the environmental impact of PHB production from lignocellulosic biomass. Fermentation contributes the most to non-renewable energy consumption, with 12,883.54 MJ of primary energy use, followed by enzymatic hydrolysis, using 9445.79 MJ, while delignification and pretreatment account for 8794.46 MJ and 7342.10 MJ, respectively. The main processes that contribute to the environmental impact of PHB production are energy use and chemical feedstock. The energy used in electricity generation plays a dominant role, particularly as the generation of electricity itself is heavily dependent on non-renewable sources (e.g., coal and natural gas). The use of chemical sources in the delignification and hydrolysis stages also adds to the environmental burden, not just from energy use but also due to the environmental effects of producing and disposing of these chemicals.

The production of 1 kg of PHB results in the generation of 345 kg of CO₂ equivalents (CO₂ eq) of carbon emissions, in comparison to the production of PLA, which generates 512.7 kg of CO_2_ eq. PHB production results in about 33% lower CO₂ emissions compared to PLA, making PHB a more environmentally favorable bioplastic in terms of its carbon footprint.

**Figure 7:**
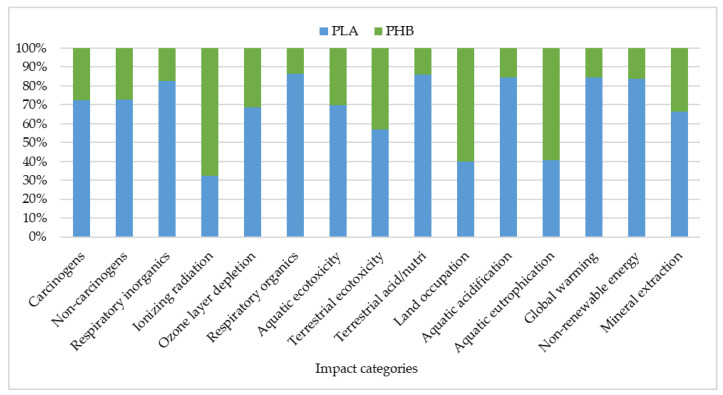
Comparative impact assessment of the PLA and PHB production.

When comparing the PLA production process to that of the PHB, it can be observed that the latter has a comparatively lower environmental impact (Figure 7).

Notable differences in the environmental impact categories are identified when analyzing the two production processes. The production of PLA from plum orchard waste has been found to account for approximately 80% of global warming, non-renewable energy, aquatic acidification, terrestrial acidification, respiratory organics, and inorganics. Meanwhile, PHB produced from apple orchard waste demonstrates a better overall toxicity profile, with lower impacts on human health (both carcinogenic and non-carcinogenic) and lower ecotoxicity compared to PLA.

Also, the production of PHB in comparison to that of PLA is associated with lower carbon emissions, reduced ecotoxicity, and a more favorable human toxicity profile. These findings reinforce the environmental benefits and potential of PHB as a sustainable bioplastic. Although PLA is currently a widely used material in the market, the environmental and health advantages of PHB could drive increased adoption in applications where sustainability and reduced toxicity are prioritized.

According to the ISO 14040 framework used, the results showed that PHB production from lignocellulosic waste has several environmental advantages over PLA, particularly in terms of carbon emissions, ecotoxicity, and human toxicity. However, energy use remains a critical factor, especially for PHB, where optimizing processes and integrating renewable energy sources can significantly improve the environmental performance.

An LCA analysis of both PLA and PHB production technologies could indicate the carbon emissions. The results presented indicate that PLA and PHB generate lower levels of greenhouse gas emissions than synthetic bioplastics. The production of 1 kg of PHB generates 345 CO_2_ eq, which is lower than the 512 kg CO_2_ eq emitted during the PLA production. The principal sources of carbon dioxide emissions associated with PLA production are biomass processing, the solid-state fermentation (SSF) process, and lactic acid purification. The production of both bioplastics results in lower carbon emissions than the manufacturing of synthetic bioplastics. However, PHB has a lower global warming potential and a smaller carbon footprint throughout its lifecycle.

The ecotoxicity of PLA and PHB production represents a significant environmental impact. The sources of ecotoxicity can be classified as either aquatic or terrestrial. The impact of aquatic ecotoxicity was 43,901.2 kg TEG water and 4871.4 kg TEG water for terrestrial ecotoxicity. In comparison, the impact for PHB production was 18,919.97 kg TEG water for aquatic ecotoxicity and 3693.62 kg TEG water for terrestrial ecotoxicity. The ecotoxicity of PHB is lower in both aquatic and terrestrial systems when compared with PLA. This is due to the pretreatment used. In pressurized hot water, the generation of waste is considerably higher than that observed in microwave irradiation. In order to minimize the environmental impact, it is recommended to optimize the pretreatment method for PLA and the fermentation process for PHB.

The technological stage employed for each bioplastic production process has an impact on the environment. In order to reduce the environmental impact, improvements and recommendations are required for each stage of the technological process. To minimize the environmental impact of the PLA production, energy consumption in the pretreatment phase must be reduced. This can be achieved through the implementation of a less energy-intensive pretreatment method. Additionally, reducing the chemical inputs used in the SSF process and employing more environmentally benign methods for lactic acid purification can further enhance sustainability. Furthermore, the polymerization phase can be made more energy-efficient by utilizing a catalyst that reacts at a lower temperature. In comparison to PLA, the production of PHB requires a reduced consumption of energy and chemicals during the delignification and enzymatic hydrolysis stages. Additionally, fermentation represents a significant source of GHG emissions. Therefore, it is advisable to utilize strains with a relatively short fermentation period to minimize the overall impact on the environment. The enhancement of waste management will facilitate the overall process efficiencies and markedly reduce the environmental impact.

The energy consumption at each stage of the production process for PLA and PHB was calculated in accordance with the non-renewable energy consumption (198,319.9 MJ) and its contribution to GHG. The pretreatment stage has the greatest impact on energy consumption (7342.1 MJ), with 65.5% of the total due to the intensive use of energy, followed by the biomass processing stage (related to the cutting, transport, and drying of the biomass), the SSF process, and the polymerization stage. The energy consumption associated with the PHB production is attributed to the processes of fermentation and enzymatic hydrolysis. The prolonged fermentation time and the energy required to maintain the controlled conditions contribute to this consumption. In conclusion, a comparative energy analysis demonstrated that PHB exhibited a reduced energy consumption due to a relatively simplified production stage, which did not involve the addition of an adjacent polymerization step.

The environmental footprint of both PLA and PHB is influenced by the use of fossil fuels. The utilization of natural gas and crude oil for heating and transportation amplifies the environmental impact. The energy-intensive pretreatment process employed in the production of PLA resulted in the consumption of five times more fossil fuel than that of PHB. Biomass processing relies on the use of natural gas, while the conversion of crude oil to plastics is enabled by the utilization of resources such as diesel, residual oil, and gasoline [38].

The obtained bioplastics (PLA and PHB) are two products that have distinct properties and sustainability benefits, and both are alternatives to conventional plastics as better sources of sustainable products. PLA is a sustainable material for biocomposites due to its biodegradability, availability, and good mechanical and thermal properties. Compared to PLA, PHB can be highly sustainable due to its environmentally friendly production, which is in line with the principles of the circular economy. It is also fully biodegradable and has high tensile strength and flexibility compared to conventional plastics, making it an ideal product for packaging and agricultural applications. Both products offer a sustainable solution for various applications and are in line with the global sustainability goals.

## 4. Conclusions

The present study employed the Life Cycle Assessment (LCA) methodology to evaluate and comparatively assess the environmental impact of two technologies, namely, the production of poly(lactic acid) (PLA) and poly(3-hydroxybutyrate) (PHB) from lignocellulosic biomass. This was conducted to select the bioplastic whose production process has the lowest environmental impact and the highest potential to contribute to the circular economy. The production process of PLA generated higher carbon emissions and had a higher ecotoxicity level compared to that of PHB. In general, the production of PHB demonstrated a relatively lower environmental impact when compared to the conversion process. However, the latter still requires optimization, given that the energy utilized in the production phase is derived from fossil fuels. Consequently, further research is necessary to develop more efficient conversion processes for biomass waste into PHB, with the objective of reducing energy consumption.

## Figures and Tables

**Table 1:** Life cycle inventory for PLA production.

Technology Stage	Process	Amount	Unit
Input			
Plum waste	Transportation and cutting	5000	kg/km
Pressurized hot water pretreatment	Water	700	L
	Cellulose	38.4	kg
	Hemicellulose	26.8	kg
	Lignin	28.6	kg
	Others (ash + protein)	6.2	kg
	Electricity (Parr reactor)	71.0	kWh
SSF process	Cellulose	31.5	kg
	Hemicellulose	1.36	kg
	Lignin	23.3	kg
	Enzymes	51.37	kg
	Nutrients and yeast	35.23	kg
	Inoculum + *L. rhamnoses*	3.52	kg
	Water	297	L
	Electricity	250	kWh
Lactic acid purification	Ammonium sulfate	10.0	kg
	Butanol	50.0	L
Polymerization and microwave	Lactic acid	29.2	kg
	SnCl_2_	11.68	kg
	o-xylene	38.54	kg
	Electricity (microwave)	100.0	kWh
Output			
Pressurized hot water pretreatment (liquid phase)	Hemicellulose	18	kg
	By-products	17	kg
	Water	600	kWh
Pressurized hot water pretreatment (solid phase)	Cellulose	31.5	kg
	Hemicellulose	1.36	kg
	Lignin	23.3	kg
SSF process	Lactic acid	29.2	L
	Waste	423.28	L
Lactic acid purification	Waste	60.0	L
Polymerization and microwave	SnCl_2_	10.2	kg
	Waste	52.62	kg
	PLA	26.8	kg

**Table 2:** Life cycle inventory for PHB production.

Technology Stage	Process	Amount	Unit
Input			
Apple waste	Transportation and cutting	5000	kg/km
Microwave irradiation	Water	700	L
	Cellulose	40.0	kg
	Hemicellulose	21.95	kg
	Lignin	29.62	kg
	Others (ash + protein)	8.43	kg
	Electricity (Parr reactor)	71.0	kWh
Delignification	Cellulose	32.2	kg
	Hemicellulose	9.18	kg
	Lignin	26.7	kg
	Ammonia	12.4	L
	Electricity	56.0	kWh
Enzymatic hydrolysis	Cellulose	25.1	kg
	Hemicellulose (trace)	0.20	kg
	Lignin (trace)	2.7	kg
	Enzymes (*T. reesei* + B-glucosidase)	29.4	kg
	Sodium citrate + water	172.19	L
	Electricity	35.2	kWh
Fermentation	Glucose	21.3	kg
	Bacterium *Bacillus megaterium*	10.3	kg
	Inoculum + water	372	kg
	Minerals	0.276	kg
	Electricity	56.2	kWh
PHB separation	PHB	10.0	kg
	Chloroform	10.0	L
Output			
Pressurized hot water pretreatment (liquid fraction)	Hemicellulose	20.0	kg
	Secondary product	9.0	kg
	Water	600	L
Pressurized hot water pretreatment (solid fraction)	Cellulose	31.5	kg
	Hemicellulose (trace)	1.36	kg
	Lignin	23.3	kg
Delignification	Lignin	22.9	kg
	Wastewater	10.0	L
Enzymatic hydrolysis	Glucose	29.2	L
	Waste	163.42	L
Fermentation	PHB	10	kg
	Waste	160	kg
PHB separation	Chloroform	10.0	L
	PHB	7.3	kg

**Table 3:** Impact categories associated with the technology stages for PLA production (I—biomass process, II—pretreatment of biomass, III—SSF process, IV—purification of lactic acid, and V—polymerization of lactic acid).

Impact Category	Unit	I	II	III	IV	V	Total
Carcinogens	kg C_2_H_3_Cl eq	1.1	2.9	0.4	0.0	0.1	4.5
Non-carcinogens	kg C_2_H_3_Cl eq	2.3	6.3	0.9	0.0	0.2	9.8
Respiratory inorganics	kg PM2.5 eq	4.7	11.4	1.3	0.0	0.1	17.5
Ionizing radiation	kBq Co-14 eq	892.5	4625.4	3142.9	5.6	1430.8	10,097.2
Ozone layer depletion	kg CFC-11 eq	0.0	0.0	0.0	0.0	0.0	0.0
Respiratory organics	kg C_2_H_4_ eq	1.6	3.8	0.4	0.0	0.0	5.7
Aquatic ecotoxicity	kg TEG water	9973.5	28,497.1	4342.8	38.8	1049.0	43,901.2
Terrestrial ecotoxicity	kg TEG soil	889.3	3033.3	690.9	7.9	250.1	4871.4
Terrestrial acid/nutrification	kg SO_2_ eq	165.6	401.7	42.6	0.1	0.5	610.4
Land occupation	m^2^org arable	0.0	1.2	0.5	0.0	0.2	1.8
Aquatic acidification	kg SO_2_ eq	22.8	55.4	6.1	0.0	0.2	84.6
Aquatic eutrophication	kg PO_4_ P-lim	0.0	0.1	0.1	0.0	0.0	0.2
Global warming	kg CO_2_ eq	3704.0	8990.5	998.3	3.3	32.8	13,728.9
Non-renewable energy	MJ primary	52,961.2	129,993.5	14,657.6	49.3	658.4	198,319.9
Mineral extraction	MJ surplus	0.3	4.1	0.9	0.1	7.2	12.5

**Table 4:** Impact categories associated with the technology stages for PHB production (I—biomass process, II—pretreatment of biomass, III—delignification, IV—enzymatic hydrolysis, V—fermentation, and VI—PHB purification).

Impact Category	Unit	I	II	III	IV	V	VI	Total
Carcinogens	kg C_2_H_3_Cl eq	0.01	0.16	0.33	0.41	0.81	0.00	1.73
Non-carcinogens	kg C_2_H_3_Cl eq	0.02	0.36	0.70	0.85	1.72	0.00	3.65
Respiratory inorganics	kg PM2.5 eq	0.05	0.64	0.81	0.89	1.30	0.00	3.69
Ionizing radiation	kBq Co-14 eq	8.92	261.24	3449.01	4878.70	12,417.18	0.00	21,015.05
Ozone layer depletion	kg CFC-11 eq	0.00	0.00	0.00	0.00	0.00	0.00	0.00
Respiratory organics	kg C_2_H_4_ eq	0.02	0.21	0.22	0.22	0.23	0.00	0.89
Aquatic ecotoxicity	kg TEG water	99.73	1609.53	3571.51	4454.37	9184.81	0.00	18,919.97
Terrestrial ecotoxicity	kg TEG soil	8.89	171.32	630.43	836.93	2046.04	0.00	3693.62
Terrestrial acid/nutrification	kg SO_2_ eq	1.66	22.69	23.68	24.13	26.51	0.00	98.67
Land occupation	m^2^org arable	0.00	0.07	0.41	0.56	1.68	0.00	2.71
Aquatic acidification	kg SO_2_ eq	0.23	3.13	3.54	3.73	4.70	0.00	15.32
Aquatic eutrophication	kg PO4 P-lim	0.00	0.01	0.05	0.07	0.19	0.00	0.32
Global warming	kg CO_2_ eq	37.04	507.79	580.04	612.45	783.51	0.00	2520.82
Non-renewable energy	MJ primary	529.61	7342.10	8794.46	9445.79	12,883.54	0.00	38,995.50
Mineral extraction	MJ surplus	0.00	0.23	1.10	1.49	3.55	0.00	6.37

## Data Availability

Data are contained within the article.
